# Progranulin deficiency causes the retinal ganglion cell loss during development

**DOI:** 10.1038/s41598-017-01933-8

**Published:** 2017-05-10

**Authors:** Yoshiki Kuse, Kazuhiro Tsuruma, Takahiro Mizoguchi, Masamitsu Shimazawa, Hideaki Hara

**Affiliations:** Molecular Pharmacology, Department of Biofunctional Evaluation, Gifu Pharmaceutical University, 1-25-4 Daigaku-nishi, Gifu, 501-1196 Japan

## Abstract

Astrocytes are glial cells that support and protect neurons in the central nervous systems including the retina. Retinal ganglion cells (RGCs) are in contact with the astrocytes and our earlier findings showed the reduction of the number of cells in the ganglion cell layer in adult progranulin deficient mice. In the present study, we focused on the time of activation of the astrocytes and the alterations in the number of RGCs in the retina and optic nerve in progranulin deficient mice. Our findings showed that the number of Brn3a-positive cells was reduced and the expression of glial fibrillary acidic protein (GFAP) was increased in progranulin deficient mice. The progranulin deficient mice had a high expression of GFAP on postnatal day 9 (P9) but not on postnatal day 1. These mice also had a decrease in the number of the Brn3a-positive cells on P9. Taken together, these findings indicate that the absence of progranulin can affect the survival of RGCs subsequent the activation of astrocytes during retinal development.

## Introduction

Astrocytes are present in the retinal nerve fiber layer (RNFL) and they secrete different types of growth factors and cytokines especially when the retina is injured. Astrocytes originate from neural precursor cells and more specifically from astrocyte precursor cells (APCs)^1, 2^. The APCs express Pax2, vimentin, and A2B5^2^, and they migrate into the retina from the optic nerve during embryogenesis and spread toward the periphery^2^. The astrocytes mature in the retina and they are mainly located in the central retina during the neonatal stage and even on postnatal day 0^2^.

The retinal ganglion cells (RGCs) are located in the ganglion cell layer (GCL) among the astrocytes, and they send their axons toward the optic nerve and to the lateral geniculate nucleus and superior colliculus^3^. The astrocytes, microglia, and oligodendrocytes in the optic nerve support and nourish the axons of the RGCs^4, 5^. APCs in the retina guide the axons toward the optic nerve during embryonic development^2^, and they are required for the normal development of the synapses of the RGCs^6, 7^.

The RGCs interact with astrocytes. For example, growth factors such as PDGF-A and sonic hedgehog are synthesized by RGCs, and they promote the migration and proliferation of APCs^2, 8, 9^. It was recently reported that an excessive activation of astrocytes induce death in cultured striatal neurons on embryonic day 18^10^. Mature astrocytes play a key role in neurogenesis during development^11^. It is believed that a balance of the activation of astrocytes is important for the normal development of neurons.

Progranulin is a multipotent growth factor that is expressed in neurons and microglia. Progranulin has been shown to promote neuronal survival and the regulation of inflammation in the brain, retina, and spinal cord^12–16^. We have shown that the retinas of adult progranulin-deficient mice, *Grn* ^*−/−*^ mice were not normal^17^. More specifically, the thickness of the outer nuclear layer (ONL) and the level of rhodopsin were significantly reduced in *Grn* ^*−/−*^ mice. In addition, we showed that recombinant progranulin promoted photoreceptor differentiation from retinal precursor cells in neonatal retinal cells in culture. We concluded that progranulin regulated normal retinal development^17^. Moreover, we confirmed that the cell number in GCL was decreased in adult *Grn* ^*−/−*^ mice although there was no change in the thickness of the inner plexiform layer (IPL), inner nuclear layer (INL) and outer plexiform layer (OPL). The purpose of this study was to determine when the alterations of the RGC and other retinal structures occur in *Grn* ^*−/−*^ mice.

## Results

### Number of Brn3a-positive retinal ganglion cells is reduced and expression of GFAP is increased in adult *Grn*^*−/−*^ mice

**Figure 1:**
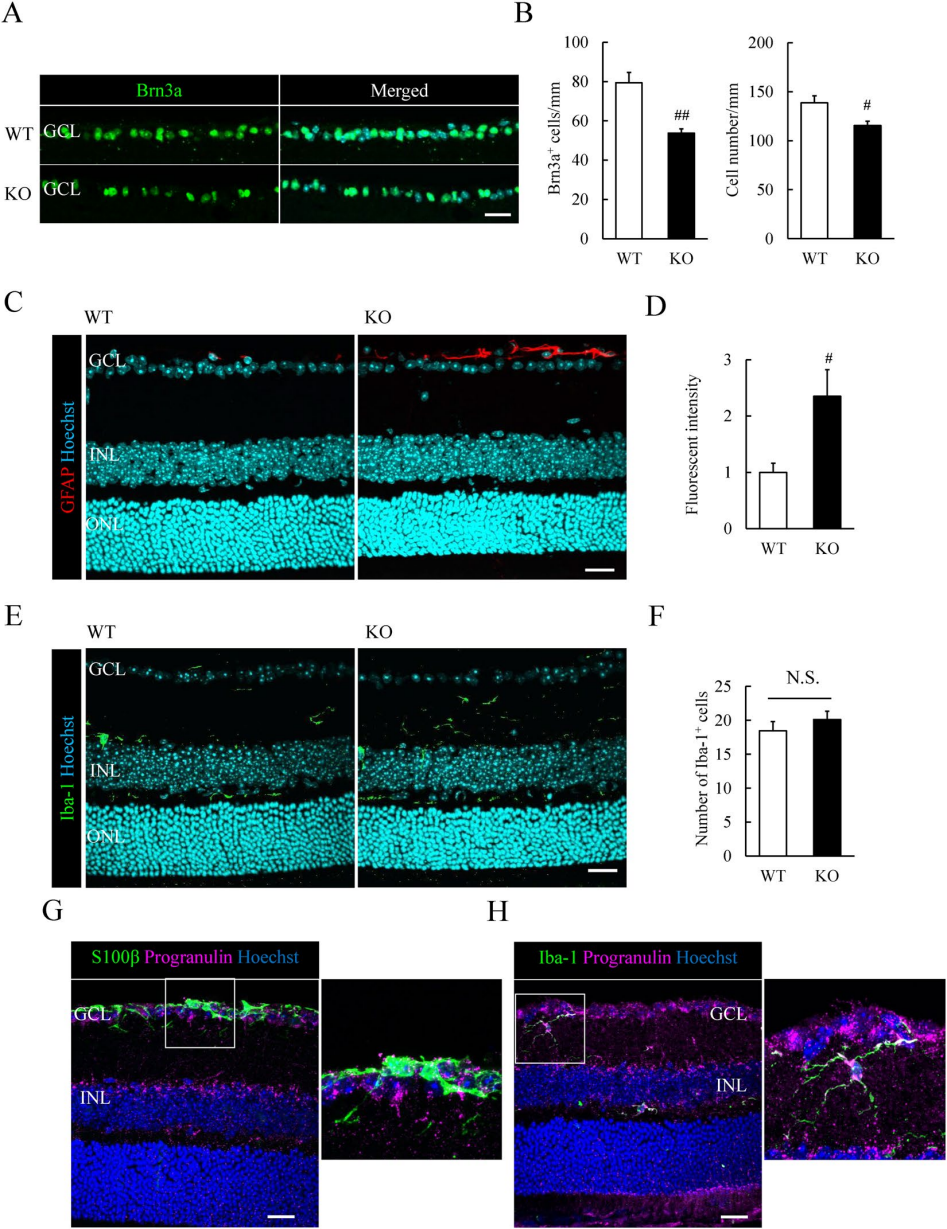
Decrease of Brn3a^+^ retinal ganglion cells (RGCs) and high GFAP expression in adult *Grn* ^*−/−*^ mice. (**A**) Brn3a staining (green) shows the RGCs in the ganglion cell layer (GCL) of wild-type (WT) and progranulin-knockout (KO) mice. Nuclei are seen as cyan by Hoechst 33342 staining. (**B**) Number of Brn3a^+^ cells and the number of cells in the GCL. The number of Brn3a^+^ cells and the cells in the GCL are reduced in *Grn* ^*−/−*^ mice. (**C**,**D**) Representative images show the activated astrocyte (red by GFAP expression) in WT and *Grn* ^*−/−*^ mice. The fluorescent intensity is increased in *Grn* ^*−/−*^ mice. (**E**,**F**) Number of Iba-1^+^ microglia (green) is not changed in *Grn* ^*−/−*^ mice. (**G**,**H**) Progranulin expression (magenta) is observed around the S100β^+^ astrocytes (green) and co-localized with Iba-1^+^ microglia (green). Data are the means ± S.E.M. (*n* = 4 or 5). ^##^ *P* < 0.01, ^#^ *P* < 0.05 vs. WT (Student’s *t*-test). WT: wild-type; KO: knock-out. Scale bar = 20 μm.

Our previous study found that the number of cells in the RNFL was reduced in *Grn* ^*−/−*^ mice^17^. To confirm whether the cell loss was due to a decrease of RGCs, we performed immunostaining for Brn3a, a specific marker for RGCs in the retina of WT and *Grn* ^*−/−*^ mice. Brn3a-positive (Brn3a^+^) RGCs and the overall number of cells in the GCL were reduced in *Grn* ^*−/−*^ mice (Fig. 1A,B).

Next, we investigated whether the glial cells were activated during retinal degeneration by staining with markers of astrocytes and microglia. GFAP is a marker of activated astrocytes^18^, and a high expression of GFAP showed that the astrocytes were activated in the *Grn* ^*−/−*^ retina. The fluorescent intensity of GFAP was significantly increased in *Grn* ^*−/−*^ retina and was higher than that in the WT retina (Fig. 1C,D). The number of Iba-1 positive (Iba-1^+^) microglia in the *Grn* ^*−/−*^ mice did not differ significantly from that in WT mice (Fig. 1E,F). Moreover, an expression of progranulin was observed around S100β-positive (S100β^+^) astrocytes and it was co-localized with Iba-1^+^ microglia (Fig. 1G,H). S100β is a marker of mature astrocytes^19^. These results demonstrated that progranulin deficiency caused the decrease in the number of RGCs and the activation of the astrocytes. Moreover, there was no change in the expression of cytokines such as tumor necrosis factor-α (TNF-α) and interleukin-1β (IL-1β) between WT and *Grn* ^*−/−*^ retina (Supplementary Fig. 1).

### Activation of astrocytes in optic nerve of *Grn*^*−/−*^ mice

**Figure 2:**
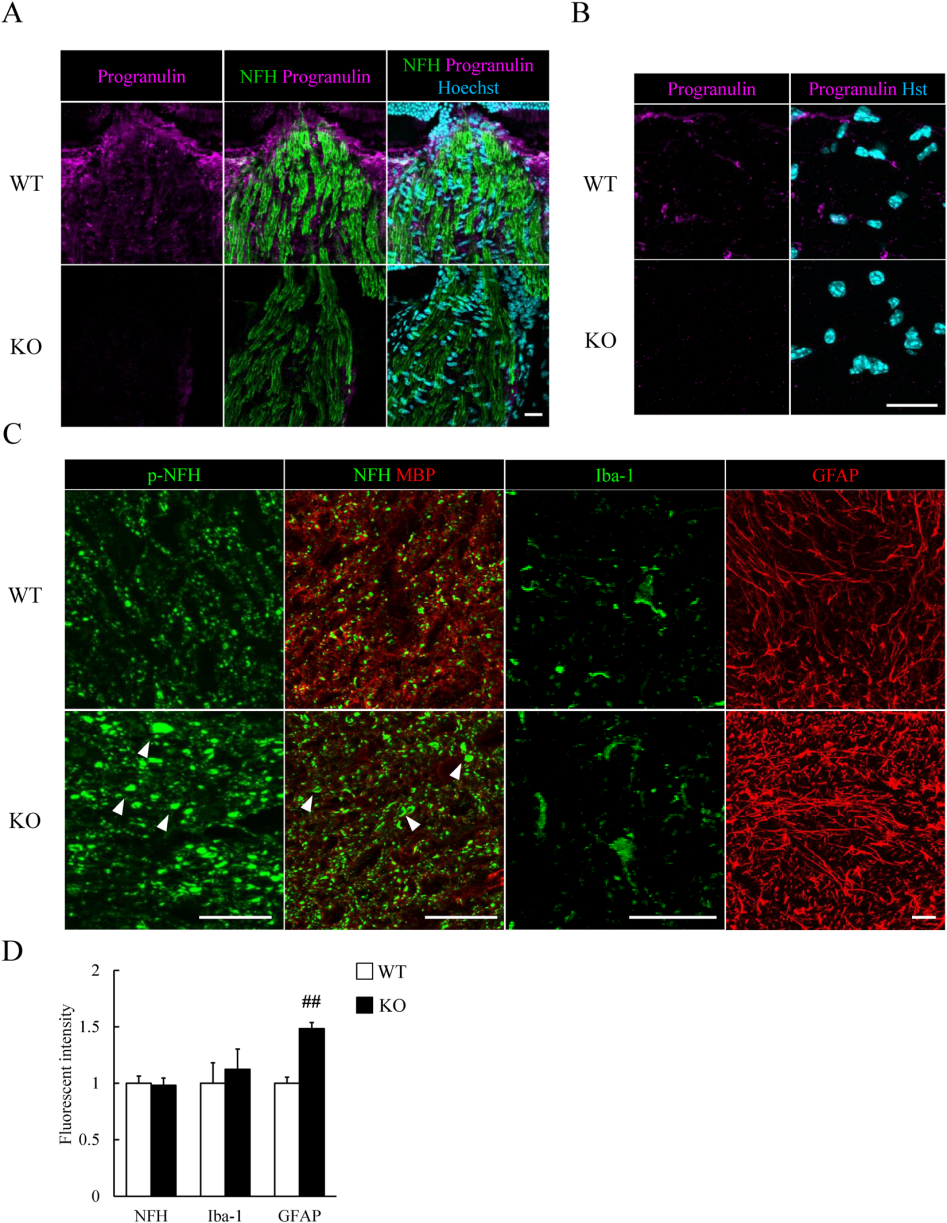
Changes of optic nerve in adult *Grn* ^*−/−*^ mice. (**A**) Progranulin expression (magenta) in WT optic nerve. Neurofilament heavy (NFH) stains the optic nerve (green). Progranulin expression is not present in optic nerve of KO mice. (**B**) Progranulin expression (magenta) in cross sections of WT and KO optic nerve. (**C**) Representative image showing phosphorylated neurofilament heavy (p-NFH), NFH, myelin basic protein (MBP), Iba-1 and GFAP. p-NFH and NFH are accumulated in *Grn* ^*−/−*^ mice. Iba-1^+^ microglia (green) is not changed in *Grn* ^*−/−*^ mice. GFAP^+^ astrocytes (red) are activated in *Grn* ^*−/−*^ mice. (**D**) The fluorescent intensity of NFH and Iba-1 is not changed. GFAP expression is increased in *Grn* ^*−/−*^ mice. Data are the means ± S.E.M. (*n* = 3). ^##^ *P* < 0.01 vs. WT (Student’s *t*-test). WT: wild-type; KO: knock-out. Scale bar = 20 μm.

An expression of progranulin was detected in longitudinal retinal sections and cross sections of the optic nerve (Fig. 2A,B). In the cross sections of the optic nerve, phosphorylated neurofilament heavy (p-NFH) and NFH were used as markers of RGC axons^5, 20^. p-NFH and NFH were accumulated in *Grn* ^*−/−*^ mice although the fluorescent intensity was not changed (Fig. 2C,D). The fluorescent intensity of Iba-1 in *Grn* ^*−/−*^ mice was not different from that in WT mice (Fig. 2C,D). Astrocytes were activated and the fluorescent intensity of GFAP was increased in *Grn* ^*−/−*^ mice (Fig. 2C,D). The optic nerve sections of *Grn* ^*−/−*^ mice showed degenerating neuronal axons and activation of astrocytes, but not microglial activation. These results indicated that there was degeneration of the RGCs in the *Grn* ^*−/−*^ retinas (Fig. 1A,B), and the axonal degeneration may precede that of the RGCs as reported in a mouse model of glaucoma^21^.

**Figure 3:**
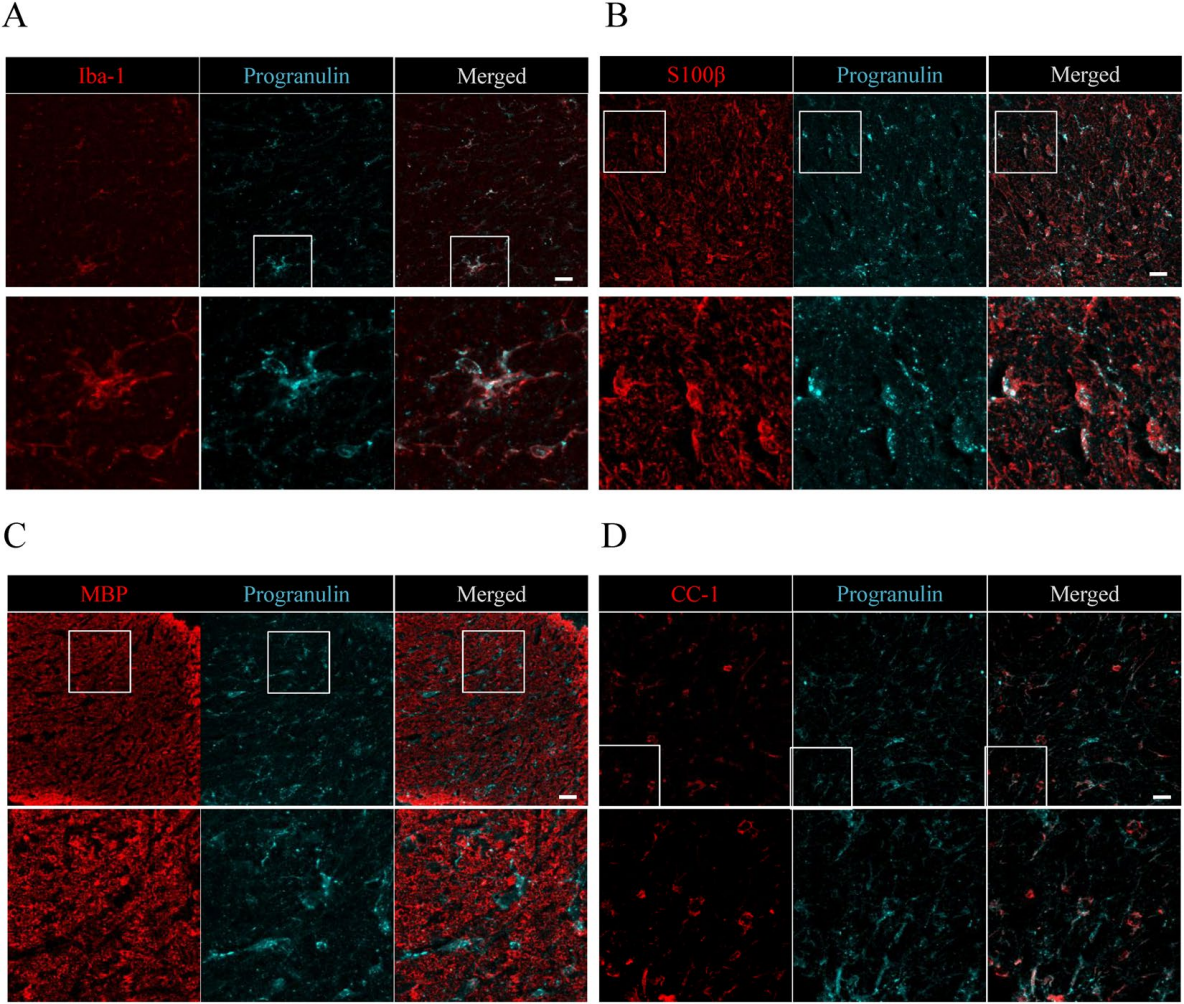
Progranulin expression in optic nerve. (**A**) Iba-1^+^ microglia (red) is co-localized with progranulin (cyan). (**B**) S100β^+^ astrocytes (red) are co-localized with progranulin. (**C**,**D**) Progranulin is partly co-localized with CC-1^+^ oligodendrocyte, but not with MBP. Scale bar = 20 μm.

An expression of progranulin was observed in Iba-1^+^ microglia, S100β^+^ astrocytes and CC-1-positive oligodendrocytes, but not in myelin basic protein (MBP)-positive myelin (Fig. 3A–D). We investigated the expression of GFAP in the hippocampus at CA1, CA3, and dentate gyrus (DG). There was no difference in the staining between the WT and *Grn* ^*−/−*^ hippocampus (Supplementary Fig. 2). We concluded that the astrocytes were activated selectively in the retina and optic nerve.

### Astrocyte activation and retinal ganglion cell loss occur in *Grn*^*−/−*^ retina on P9

**Figure 4:**
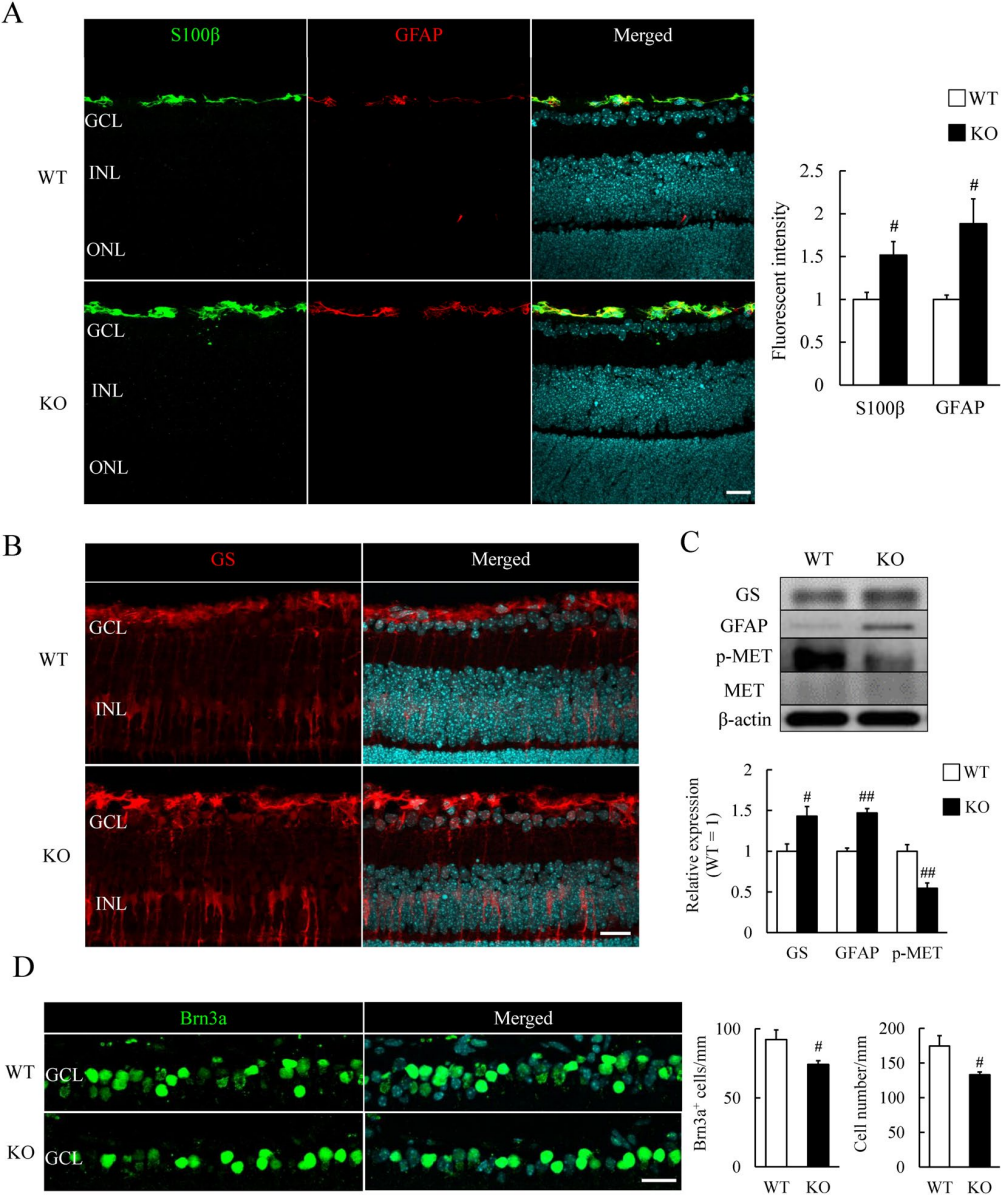
Retinal astrocyte activation and retinal ganglion cell loss in *Grn* ^*−/−*^ mice at P9. (**A**) S100β^+^ and GFAP^+^ astrocytes (green and red) in P9 mice retina. Fluorescent intensity of S100β and GFAP expression is increased in *Grn* ^*−/−*^ mice. Data are the means ± S.E.M. (*n* = 5 or 6). ^#^ *P* < 0.05 vs. WT (Student’s *t*-test). (**B**) GS (red) stained astrocytes and Müller glia. Astrocytes activation is observed in *Grn* ^*−/−*^ mice at P9. (**C**) Typical band showed GS, GFAP, p-MET, MET, and β-actin. The quantitative data show the relative expression of GS and GFAP per β-actin and p-MET per MET. GS and GFAP expressions are increased in *Grn* ^*−/−*^ mice retina and the phosphorylation of MET is decreased in *Grn* ^*−/−*^ mice retina at P9. Data are the means ± S.E.M. (*n* = 3 to 5). ^##^ *P* < 0.01, ^#^ *P* < 0.05 vs. WT (Student’s *t*-test). (**D**) Brn3a staining (green) shows the RGCs. The nuclei are stained cyan by Hoechst 33342. The quantitative data show that the number of Brn3a^+^ cells and the cell numbers in the GCL were decreased in *Grn* ^*−/−*^ mice at P9. Data are the means ± S.E.M. (*n* = 5). ^#^ *P* < 0.05 vs. WT (Student’s *t*-test). WT: Wild-type; KO: Knock-out. Scale bar = 20 μm. The cropped blots are used in this Figure and the full-length blots are presented in Supplementary Fig. S4.

To determine when the number of RGCs is decreased and astrocytes are activated, we first examined the expression of S100β and GFAP, respectively, in P9 retinas. The fluorescent intensity of S100β and GFAP expression was increased in *Grn* ^*−/−*^ RNFL and western blotting showed an increase of GFAP expression (Fig. 4A,C). Moreover, the expression of glutamine synthetase (GS), another glial marker, was increased in the *Grn* ^*−/−*^ RNFL and INL consisting of astrocytes and Müller glia respectively (Fig. 4B,C).

The results of our previous study showed that progranulin was associated with the hepatocyte growth factor (HGF) receptor which is also called MET^15, 17^. We confirmed that progranulin deficiency strongly inhibited the phosphorylation of the HGF receptor (Fig. 4C). At this stage, the number of Brn3a^+^ RGCs and the cell number in GCL were reduced in *Grn* ^*−/−*^ mice. These results demonstrated that astrocytes and RGCs were already affected at P9. Another finding suggested that progranulin deficiency inhibited the maturation of photoreceptors from the photoreceptor precursor cells at P9. More specifically, the expression of CRX was increased and the rhodopsin expression was decreased in the *Grn* ^*−/−*^ retina at P9 (Supplementary Fig. 3). These results are a complementary findings of the earlier report that progranulin promoted the differentiation of photoreceptors during development^17^.

### Progranulin does not affect astrocytes in *Grn*^*−/−*^ retina on P1

**Figure 5:**
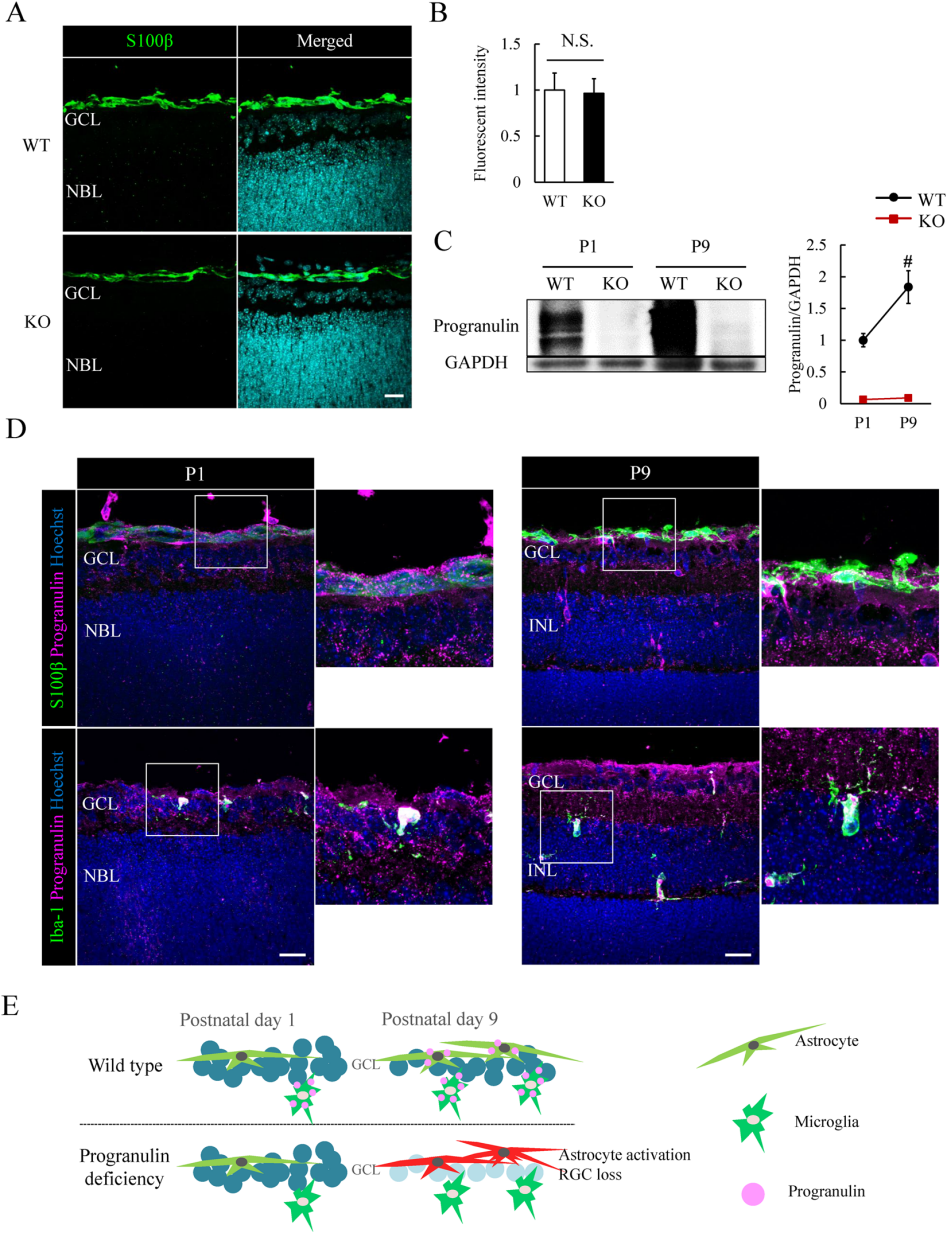
No change in retinal astrocytes in *Grn* ^*−/−*^ mice at P1. (**A**,**B**) Representative image show the S100β^+^ astrocytes (green) in P1 mice retina. There is no change in the expression of S100β in *Grn* ^*−/−*^ mice at P1. Data are the means ± S.E.M. (*n* = 4, 5). Student’s *t*-test. (**C**) Progranulin expression in P1 and P9 mice retina by western blotting. Progranulin expression is not observed in *Grn* ^*−/−*^ mice at P1 and P9 but is expression in WT mice at both times. The expression of progranulin is higher at P9 than at P1 in WT mice retina. Data are the means ± S.E.M. (WT: *n* = 3 or 4, KO: *n* = 2). ^#^ *P* < 0.05 vs. P1 WT (Student’s *t*-test). (**D**) Progranulin expression (magenta) in P1 and P9 WT mice retina by immunostaining. S100β^+^ astrocyte (green) is partly co-localized with progranulin in P9 WT retina but not in P1 WT retina. Iba-1^+^ microglia (green) are almost fully co-localized with progranulin in P1 and P9 retina. (**E**) Retinal development with or without progranulin. Progranulin is mainly secreted by microglia. Progranulin deficiency leads to astrocyte activation and RGC loss at P9, but not at P1. WT: Wild-type; KO: Knock-out; N.S.: Not significance. Scale bar = 20 μm. The cropped blots are used in this Figure and the full-length blots are presented in Supplementary Fig. S4.

Finally, we investigated the changes of the astrocyte in the *Grn* ^*−/−*^ retina at P1 to determine whether progranulin deficiency affected the gliogenesis at this time. There was no change in the expression of S100β in the central retina of *Grn* ^*−/−*^ mice on P1 (Fig. 5A,B). An expression of GFAP was rarely observed at P1 in the retina of WT and *Grn* ^*−/−*^ mice (data not shown). The expression of progranulin was investigated at P1 and P9 by western blotting in the retina of WT and *Grn* ^*−/−*^ mice. Progranulin expression was not detected in *Grn* ^*−/−*^ mice at P1 or P9 (Fig. 5C). The WT retinas at P9 expressed progranulin higher than at P1 in WT retinas (Fig. 5C). Moreover, immunostaining showed that progranulin was localized in the retinas at P1 and P9 of WT mice. S100β^+^ astrocytes were partly co-localized with progranulin in P9 WT retinas, but not with P1 WT retinas (Fig. 5D). Iba-1^+^ microglia was almost fully co-localized with progranulin in P1 and P9 retina (Fig. 5D). In normal development, progranulin expression was increased from P1 to P9. RGCs and astrocytes strongly depended on the presence of progranulin during this stage (Fig. 5E). We suggest that progranulin is associated with the survival of RGCs during retinal development (P1 to P9).

## Discussion

The maturation of RGCs is completed soon after birth^22^. In the present study, the astrocytes have not been activated and the number of astrocytes in *Grn* ^*−/−*^ mice does not differ significantly from that in WT mice at P1 (Fig. 5A,B). This indicates that the astrocytes probably do not affect the development of the retinal neurons until at least P1 in *Grn* ^*−/−*^ mice. To support this hypothesis, the results of this study showed that the expression of the progranulin protein was upregulated in WT retina from P1 to P9 (Fig. 5C). Thus, progranulin may not be absolutely necessary for the normal development of the retina up to P1.

The phosphorylation of HGF receptors is reduced in *Grn* ^*−/−*^ mice at P9 (Fig. 4C). From this observation and previous reports, it is highly likely that progranulin is associated with the HGF receptor^15, 17, 23^. More specifically, a decrease of phosphorylation in the *Grn* ^*−/−*^ retina and an increase of the phosphorylation in primary retinal cells by recombinant progranulin were observed in the earlier and present studies. These changes should result from the activity of retinal neuronal cells and, not by only astrocytes considering the abundance of astrocytes in the retina. It has been reported that *c-met*, a HGF receptor gene, was present in RGCs and exogenous HGF protected RGCs through HGF receptor signaling^24, 25^. Therefore, progranulin may contribute to the direct protection of RGCs through HGF receptor signaling at immature stage followed by the activation of astrocytes.

While, it was recently reported that excessive activation of astrocytes induces the death of neuronal cells in culture^10^. In the present study, astrocyte markers, S100β, GFAP, and GS were up-regulated in *Grn* ^*−/−*^ mice at P9 (Fig. 4A–C). The expression of GFAP probably indicates that the astrocytes are activated^18, 19^, and the activation was occurred at P9 in *Grn* ^*−/−*^ mice (Fig. 4A). Recently, it was reported that recombinant progranulin directly suppresses the activation of astrocytes using murine primary astrocytes^26^. Therefore, the excessive activation of astrocytes in *Grn* ^*−/−*^ retina might have caused the loss of Brn3a^+^ RGCs (Fig. 4D). It was believed that astrocyte activation and RGCs degeneration are sustained at the adult stage, which then results in axonal degeneration (Figs 1A–D and 2C).

An expression of progranulin was observed in the microglia at P1, P9 and adult retinas and optic nerves (Figs 1H, 3A and 5D). This is reasonable considering previous reports that progranulin is mainly secreted by microglia and neurons in the brain during early and adult ages^16, 27, 28^.

Our immunostaining data showed that the S100β^+^ astrocytes were partly co-localized with progranulin positive cells (Figs 1G, 3B and 5D). This is generally not observed in the brain although it was reported that some of the astrocytes in the brain express progranulin^28^. This could be due to the differences in the astrocyte markers, S100β as we used GFAP as previously performed. Although it is not certain whether astrocytes secrete progranulin, we think the possibility that the progranulin surrounding the astrocytes may play a role on astrocytes at P9 and also of adult retinas and optic nerve (Figs 1C, 2C and 4A).

The expression of progranulin has been detected in the brain, spinal cord, and retinal ganglion cells during development^29, 30^. It was later reported that a mutation of the progranulin gene was associated with the frontotemporal lobar degeneration^31^. However, the role played by progranulin during the development of the CNS has not been fully determined. The results of this study showed that progranulin played a key role in the survival of RGCs. These findings indicate that progranulin plays important roles during development of the retina.

## Materials and Methods

### Animals

Adult C57BL/6 mice (Japan SLC, Hamamatsu, Japan) and neonatal mice were maintained under controlled lighting environment (12 h/12 h light/dark cycle). *Grn* ^*−/−*^ mice generated by Kayasuga *et al*.^32^ were obtained from Riken BioResource Center (Tsukuba, Japan) and were backcrossed with C57BL/6 mice. All experiments were performed in accordance with the ARVO Statement for the Use of Animals in Ophthalmic and Vision Research, and the procedures were approved and monitored by the Institutional Animal Care and Use Committee of Gifu Pharmaceutical University.

### Preparation of brain, eye, and optic nerve tissues

Mice were anesthetized with an intraperitoneal injection of 50 mg/kg sodium pentobarbital (Nacalai Tesque, Inc., Kyoto, Japan), perfused with saline and then immediately perfused with 4% paraformaldehyde (PFA) for 8 min. The brain, eye, and optic nerve were removed and further fixed in 4% PFA for 24 h at 4 °C. The tissues were immersed in 25% sucrose solution for 24 h at 4 °C and frozen in optimal cutting temperature compound (Sakura Finetechnical Co., Ltd., Tokyo, Japan). Finally, the brain (12 μm) and eye and optic nerve (10 μm) were sectioned and mounted on glass slides (MAS COAT; Matsunami Glass Ind., Ltd., Osaka, Japan).

### Immunostaining

Immunostaining was performed according to the methods described in detail^17^. Briefly, the sections were blocked by non-immune serum and incubated overnight with the primary antibody at 4 °C. The mouse-on-mouse (M.O.M) immunodetection kit (Vector Labs, Burlingame, CA, USA) was used for blocking and solvents. After an overnight incubation with the primary antibody, the sections were incubated with the secondary antibody for 1 h. They were then counterstained and mounted.

For the Brn3a staining, the retinal sections were pre-treated with 0.1% trypsin (Wako Pure Chemical Industries, Ltd., Osaka, Japan) at 37 °C for 10 min and incubated with the Brn3a antibody [1:50 dilution: SantaCruz (Dallas, Texas, USA)] for 1 h at 37 °C.

The following primary antibodies were used: mouse anti-rhodopsin (1:1000 dilution: Millipore, Bedford, MA, USA), mouse anti-GS (1:1000 dilution: Millipore), mouse anti-GFAP (1:1000 dilution: SantaCruz), mouse anti-NFH (1:1000 dilution: Millipore), mouse anti-p-NFH (1:1000 dilution: Millipore), mouse anti-MBP (1:200 dilution: Millipore), mouse anti-CC-1 (1:100 dilution: Millipore), rabbit anti-Iba-1 (1:50 dilution: Wako), rabbit anti-S100β [1:200 dilution: Abcam (Cambridge, MA, USA)], rabbit anti-NFH (1:1000 dilution: Millipore), rabbit anti-CRX (1:20 dilution: SantaCruz), sheep anti-progranulin (1:5 dilution: R&D Systems, Minneapolis, MN, USA), goat anti-TNF-α (1:20 dilution: SantaCruz), rabbit anti-IL-1β (1:100 dilution: Abcam), Alexa Fluor^®^488 goat anti-mouse IgG, Alexa Fluor^®^488 goat anti-rabbit IgG, Alexa Fluor^®^488 donkey anti-goat IgG, Alexa Fluor^®^546 goat anti-mouse IgG, Alexa Fluor^®^546 donkey anti-rabbit IgG, and Alexa Fluor^®^647 donkey anti-sheep IgG (Invitrogen, Carlsbad, CA, USA).

Images were acquired with a confocal microscope (FLUOVIEW FV10i; Olympus, Tokyo, Japan). For quantitative data, photographs were taken at 500 µm from the optic nerve head (P9 and adult retina) and at the central retina (P1 retina). The fluorescent intensity was measured by ImageJ (National Institutes of Health, Bethesda, MD). The Brn3a^+^ cells were counted within the area of the image (211.968 × 211.968 µm). The density is presented as the number/mm. The number of Iba-1^+^ cells were counted within whole retinal sections.

### Western blotting analysis

Western blotting was performed according to our methods described in detail^17^. Briefly, mice retinas were lysed using a buffer containing inhibitors of protease and phosphatase. The tissue was homogenized and the cell lysate was centrifuged. The supernatant was used for the subsequent experiments. The protein concentration was measured using a protein assay kit (Thermo Scientific, Waltham, MA, USA). Samples were analyzed by SDS-PAGE using 5–20% gradient gels (Wako), and the proteins were transferred onto a membrane. After blocking for 30 min at room temperature, the membranes were washed and then incubated with the primary antibody overnight at 4 °C. The following primary antibodies were used: mouse anti-rhodopsin (1:1000 dilution: Millipore), mouse anti-GS (1:1000 dilution: Millipore), mouse anti-GFAP (1:1000 dilution: SantaCruz), mouse anti-MET [1: 500 dilution, Cell Signaling Technology (Danvers, MA, USA)], rabbit anti-CRX (1:100 dilution: SantaCruz), rabbit anti-p-MET (1: 1000 dilution, Cell Signaling Technology), sheep anti-progranulin (1:100 dilution: R&D systems), and mouse anti-β-actin (1:2000 dilution: Sigma-Aldrich). After exposure to the primary antibody, the membranes were incubated with peroxidase goat anti-rabbit, goat anti-mouse or rabbit anti-sheep IgG (Thermo Scientific) as the secondary antibody. The immunoreactive bands were made visible with ImmunoStar LD (Wako).

### Statistical analysis

The data are presented as the means ± standard error of the means (SEMs). The significance of the differences were determined by two-tailed Student’s *t*-test [STAT VIEW version 5.0 (SAS Institute, Cary, NC, USA)]. A *P* < 0.05 was considered statistically significant.

## Electronic supplementary material

Supplementary Info
