# Supplementary material for: Progranulin deficiency causes the retinal ganglion cell loss during development
Source: Sci Rep. 2017 May 10;7:1679. doi: 10.1038/s41598-017-01933-8 (PMC5431873; doi:10.1038/s41598-017-01933-8)
Supplement: Supplementary file 1 — Supplementary Info [file 41598_2017_1933_MOESM1_ESM.pdf]

Progranulin deficiency causes the retinal ganglion cell loss during development.

Yoshiki Kuse, Kazuhiro Tsuruma, Takahiro Mizoguchi, Masamitsu Shimazawa, Hideaki Hara*

Molecular Pharmacology, Department of Biofunctional Evaluation, Gifu Pharmaceutical University, 1-25-4 Daigaku-nishi, Gifu 501-1196, Japan.

*For reprints and all correspondence: Professor H. Hara, Ph.D., R.Ph., Molecular Pharmacology, Department of Biofunctional Evaluation, Gifu Pharmaceutical University, 1-25-4 Daigaku-nishi, Gifu 501-1196, Japan.
Email: hidehara@gifu-pu.ac.jp

Supplementary information

Supplementary Methods

Immunostaining

The sections of brains and retinas were prepared as described in the main text. Then, immunostaining was performed as described in the main text.

Western blotting analysis

Western blotting was performed according to described methods in the main text.

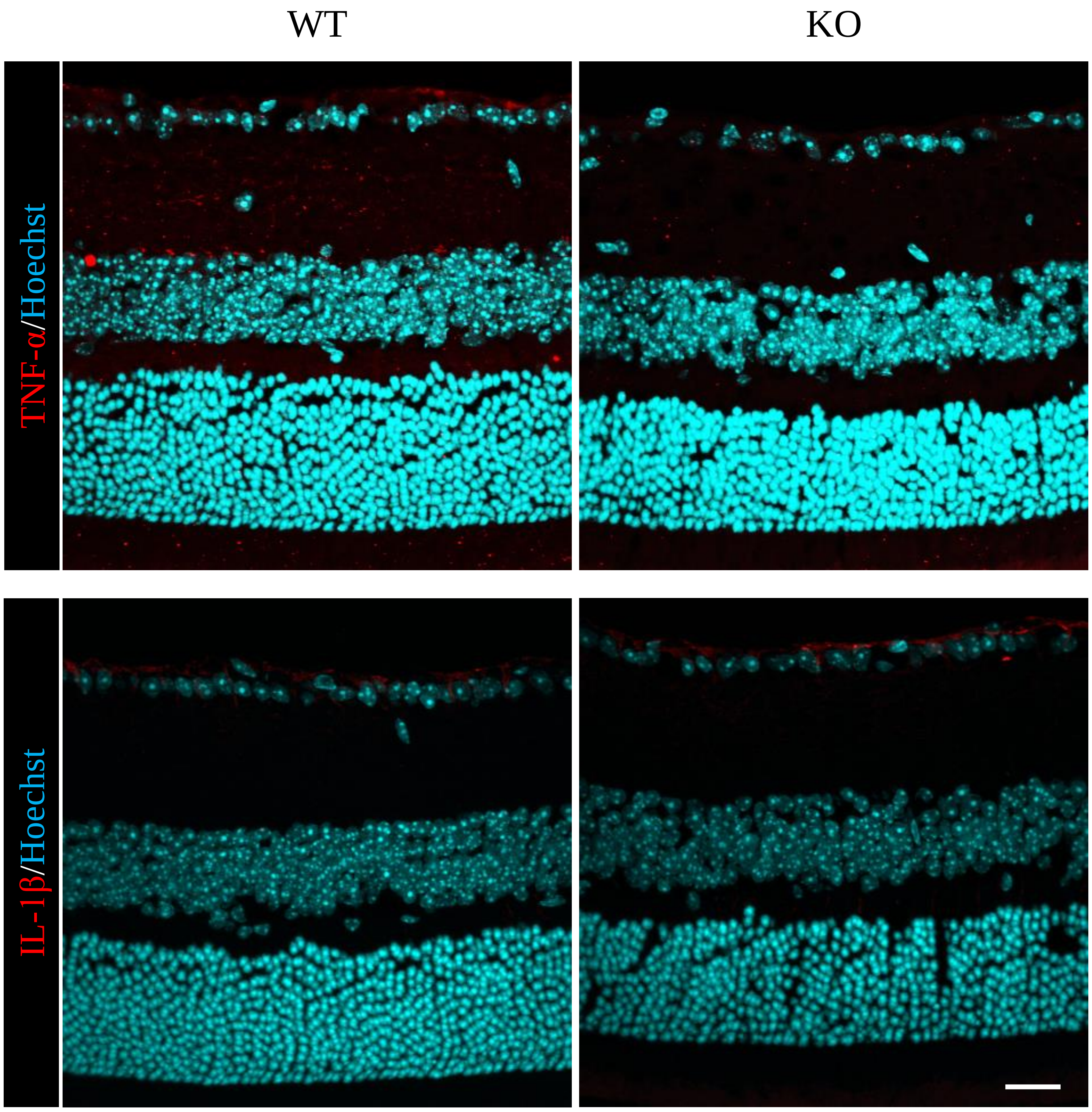

Figure S1 No change of expression of cytokines in *Grn*$^{-/-}$ mice.
Typical image showed the expression of TNF-α and IL-1β. There is no change between WT and KO mice. Data are the means ± S.E.M. (n = 3), WT: Wild-type; KO: Knock-out. Scale bar = 20 μm.

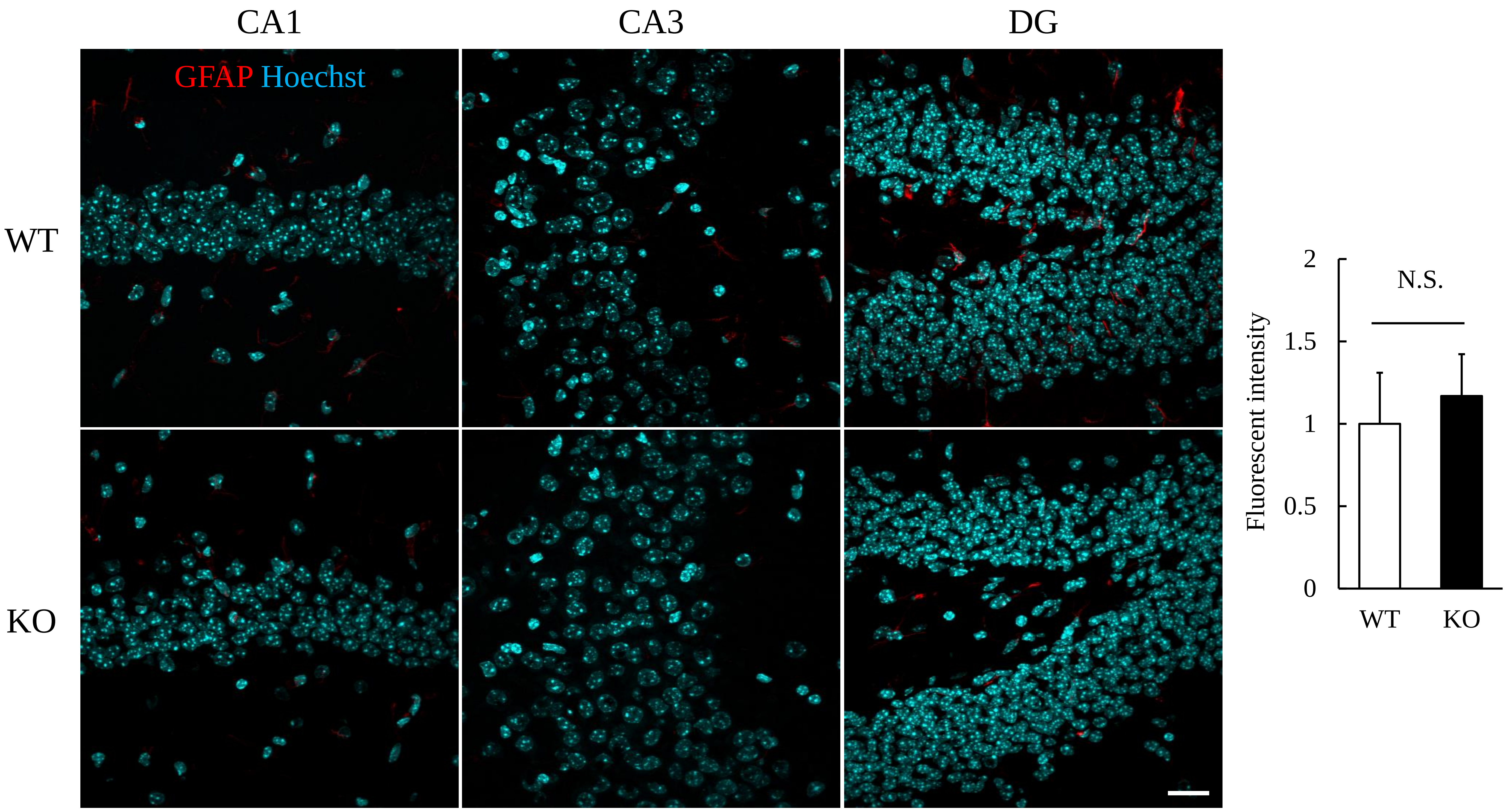

Figure S2 No change of GFAP expression in adult *Grn*$^{-/-}$ mice hippocampus.
Typical image showed the GFAP expression in CA1, CA3, and DG. There is no change between WT and KO mice. Data are the means ± S.E.M. (n = 4 or 5), Student's t-test. WT: Wild-type; KO: Knock-out. Scale bar = 20 μm.

Supplementary Figure 3

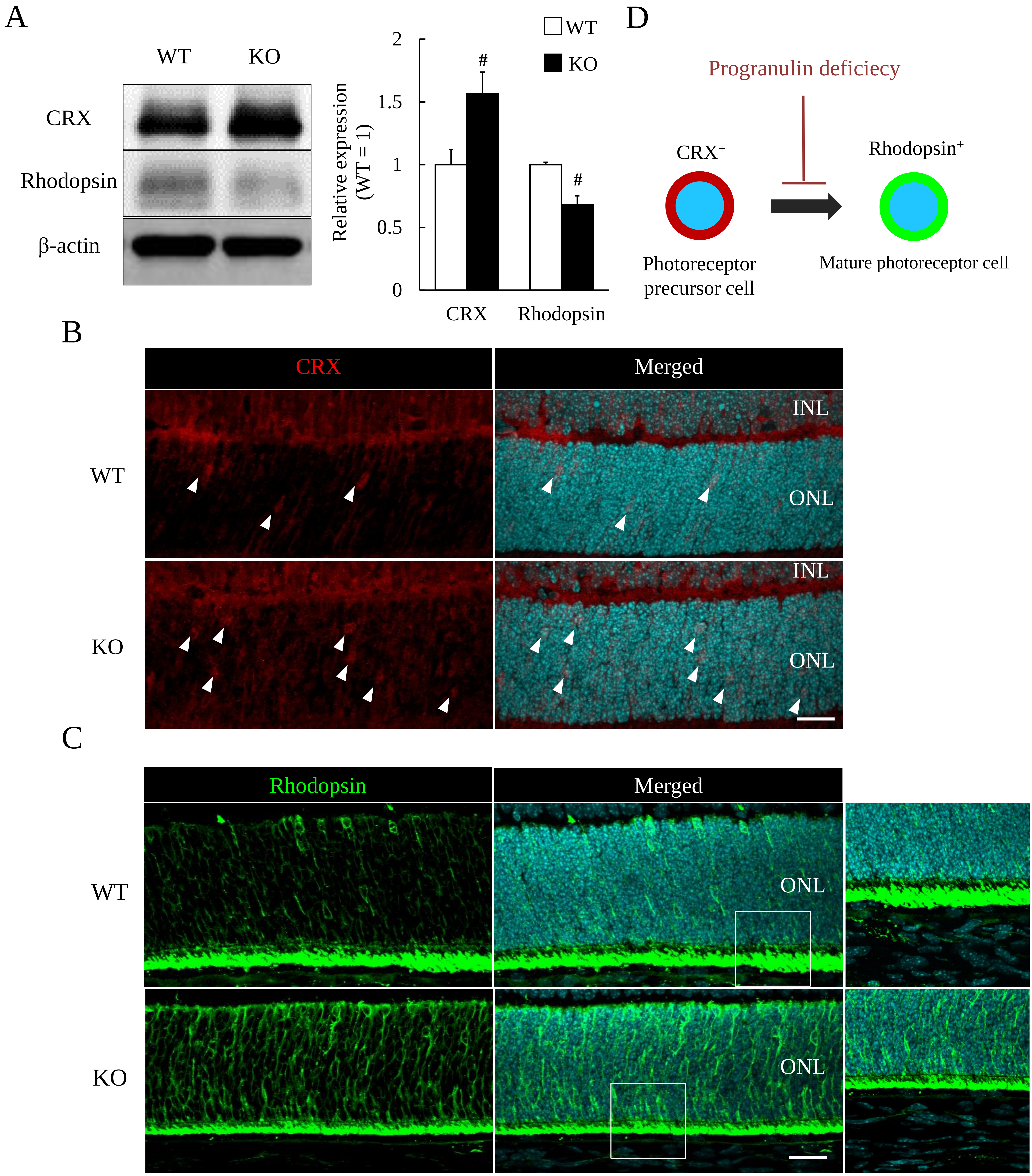

Figure S3 The inhibition of photoreceptor development in *Grn*$^{-/-}$ mice at P9.
(A-C) Typical image showed the expression of CRX and rhodopsin by western blotting and immunostaining. CRX expression was increased and rhodopsin expression was decreased in *Grn*$^{-/-}$ mice at P9. (D) PGRN deficiency inhibited the differentiation of CRX^{+} photoreceptor precursor cells to rhodopsin^{+} mature photoreceptor cells. Data are the means ± S.E.M. (n = 3 or 4). $^{\#}$; $P < 0.05$ vs.WT (Student's *t*-test). WT: Wild-type; KO: Knock-out. Scale bar = 20 μm. The cropped blots are used in this Figure and the full-length blots are presented in Supplementary Figure S4.

Supplementary Figure 4

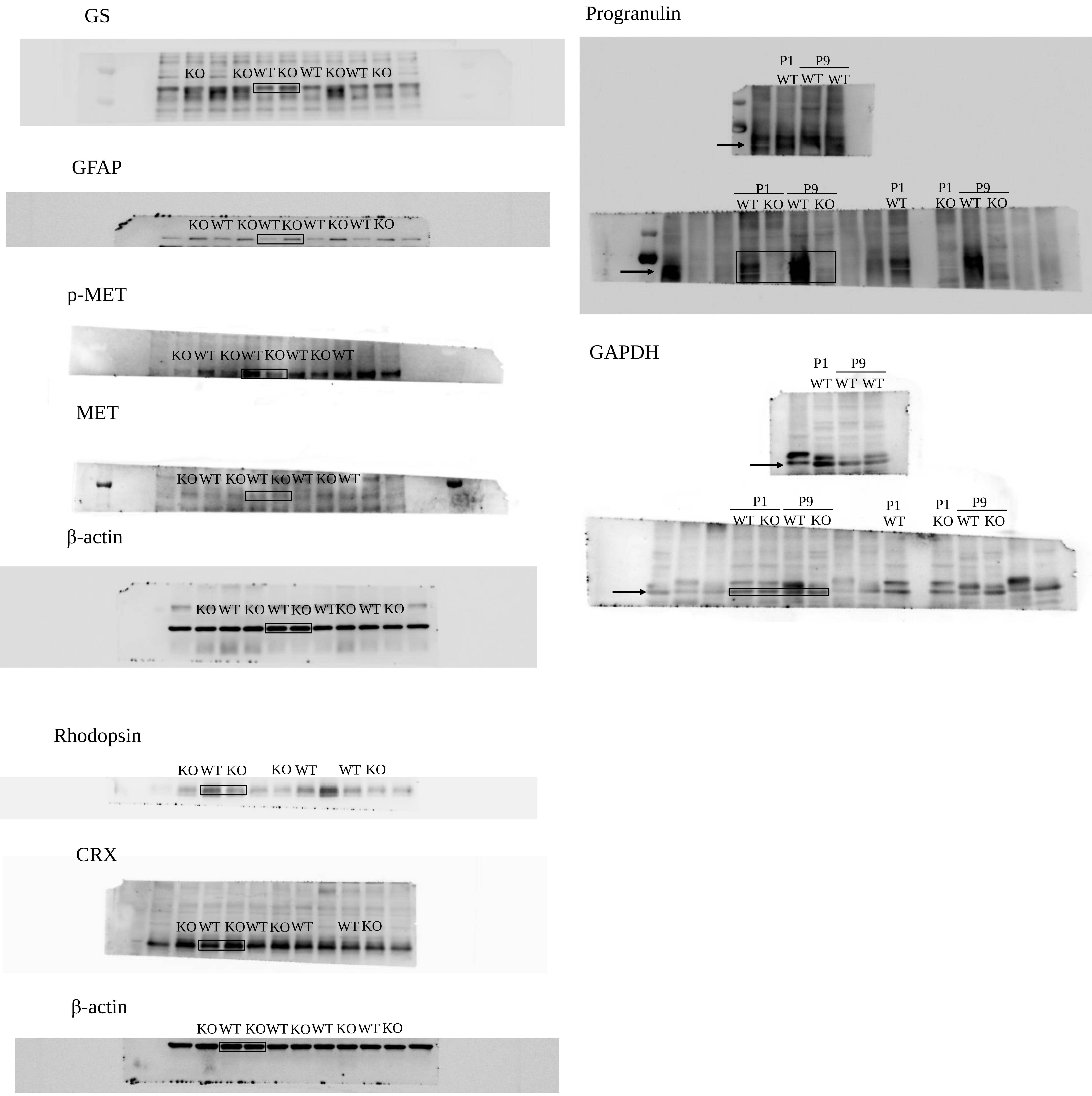

Figure S4 The full-length blots of results by western blotting.
